# Supplementary material for: Lipid-Based Nanoparticles Fused with Natural Killer Cell Plasma Membrane Proteins for Triple-Negative Breast Cancer Therapy
Source: Pharmaceutics. 2024 Aug 29;16(9):1142. doi: 10.3390/pharmaceutics16091142 (PMC11434974; doi:10.3390/pharmaceutics16091142)
Supplement: Supplementary file 1 [file pharmaceutics-16-01142-s001.zip › pharmaceutics-3155162-supplementary.pdf]

Supplementary Materials

Lipid-based nanoparticles fused with natural killer cell plasma membrane proteins for triple-negative breast cancer therapy

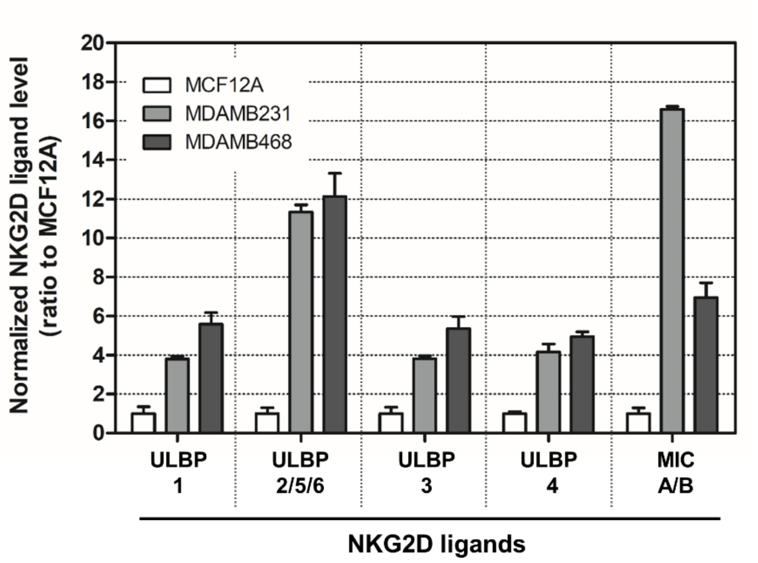

Figure S1. Comparing NKG2D ligands expression level of normal breast cell and TNBC cells. FACS analysis data for NKG2D ligands (ULBP1, ULBP2/5/6, ULBP3, ULBP4 and MICA/B) expression confirmation in normal breast cell (MCF12A) and TNBC cell lines (MDA-MB-231 and MDA-MB-468).

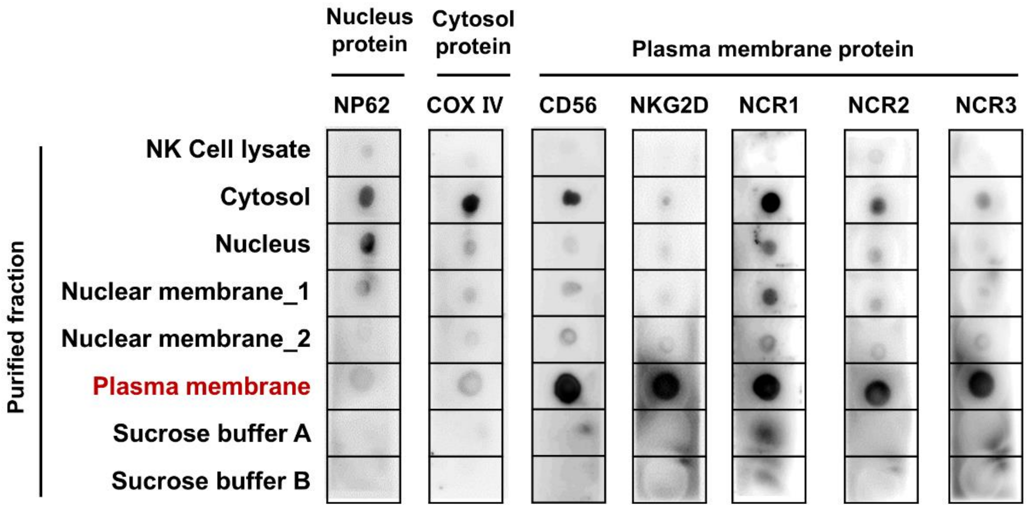

Figure S2. Purification of plasma membrane proteins from NK92 cells. Immunoblotting of isolated NK cell membrane proteins to identify fractions enriched only in membrane proteins: NP62; nuclear protein, COX IV; cytosolic protein, CD56; NK cell plasma membrane protein marker, NKG2D; NCR1(NKp46), NCR2 (NKp44), and NCR3(NKp30); and NK cell killer activating receptor.

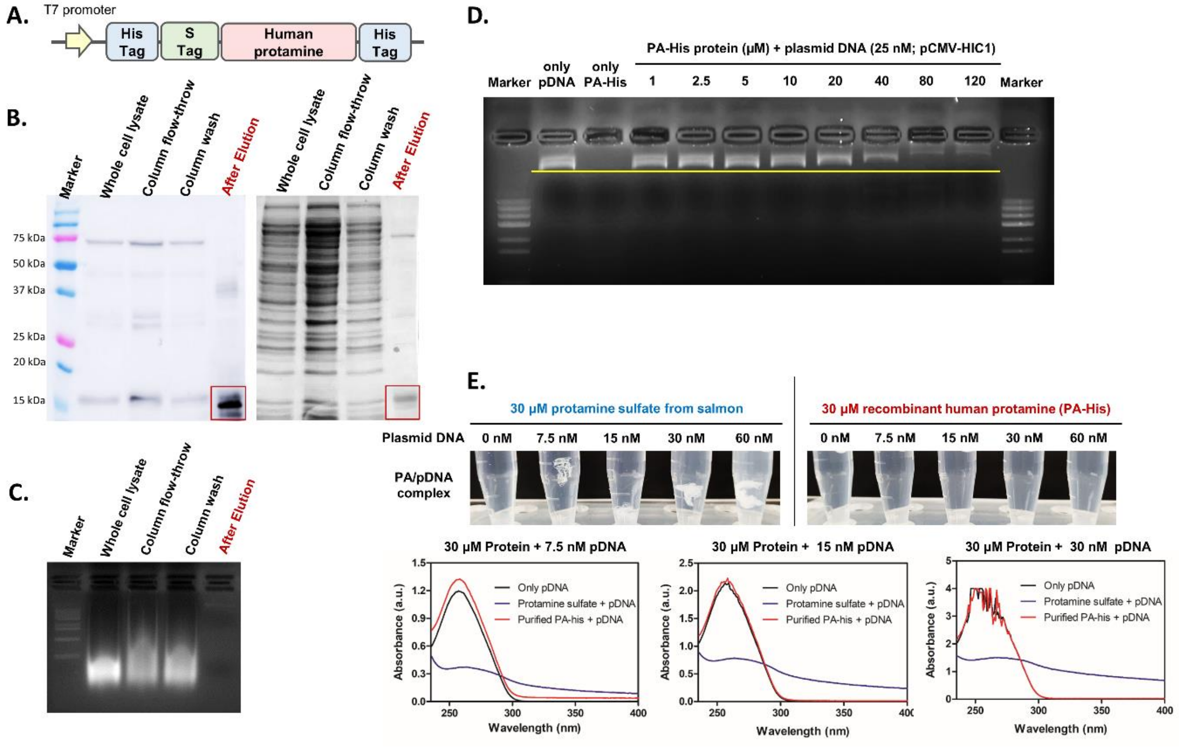

Figure S3. Preparation of recombinant human protamine. (A) Sequence scheme of recombinant human protamine (PA) protein. (B) Purification of recombinant PA proteins from *E. coli* and determination by SDS-PAGE and western blot. (C) *E.coli* RNA contamination measurement of purified PA protein. (D) Complexation of modified PA protein and *HIC1* plasmid DNA. The plasmid DNA (1.5 nM; 10 kbp) was treated with the modified PA protein (1, 2.5, 5, 10, 20, 40, 80, and 120 μM) in PBS buffer (10 μL) at 37 °C for 1 h. Products were resolved on a 1 % agarose gel. (E) Complex solution stability assessment of the modified PA and plasmid DNA in PBS. The plasmid DNA (0, 7.5, 15, 30, and 60 nM) was mixed with the unmodified protamine sulfate or PA solution (20 μM) in PBS (pH 7.2, 1 mL) at 25 °C for 5 min. Products were observed in a UV-visible spectrometer.

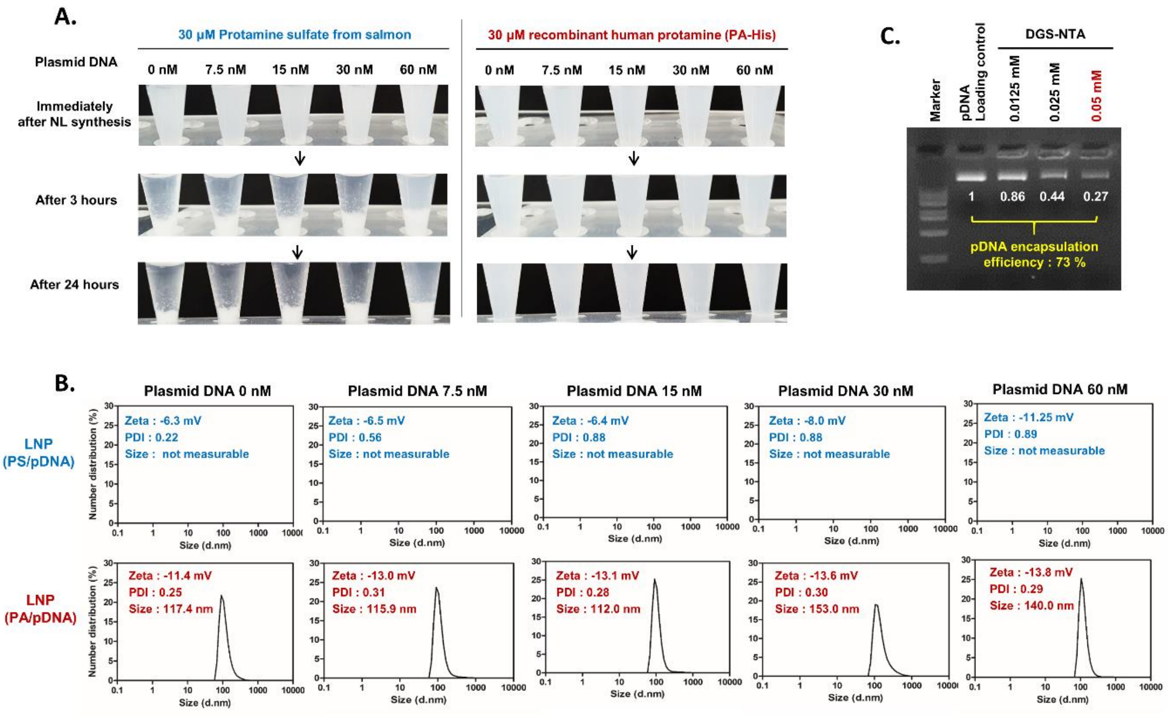

Figure S4. Solution stability of LNP after encapsulation of PA/pDNA complexes. (A) LNP stability of the modified PA/pDNA and protamine sulfate (PS)/pDNA in PBS. Plasmid DNA (0, 7.5, 15, 30, and 60 nM) was treated with the PA (20 μM) and PS (20 μM) solution in PBS buffer (1 mL) at 37 °C for 1 h. The lipid film was hydrated with the PA/pDNA complex dispersed in 1 mL PBS solution. After the freeze-thawing procedure, the LNP(PA/pDNA) solution was incubated at 25 °C. (B) LNP size distribution data with the PA /pDNA and PS/pDNA in PBS after 24 h. (C) Encapsulation optimization of PA/pDNA at 0.0125, 0.025, or 0.05 mM of DGS-NTA-Ni.

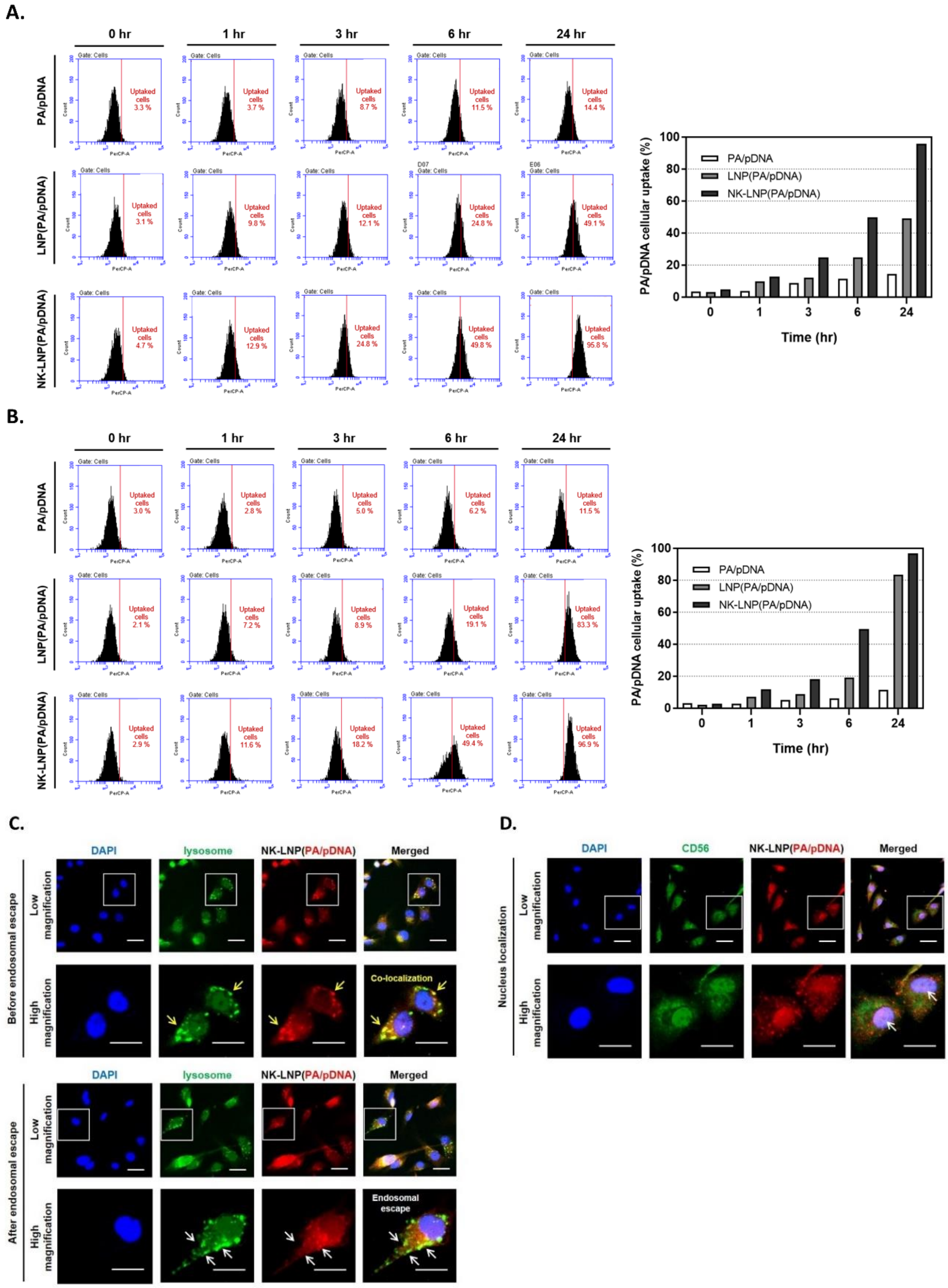

Figure S5. FACS and fluorescence images of PA/pDNA cellular uptake, endosomal escape, and nucleus localization after treatment of TNBC. RITC-labelled PA/pDNA containing NK-LNP(PA/pDNA) was used to treat MDA-MB-231 (A) and MDA-MB-468 (B) at different time points. Cells were fixed and analyzed, and the results were compared with PA/pDNA alon and LNP(PA/pDNA) without NK cell membrane protein fusion. The low magnification and color channel separation data for Fig 2C (C) and 2D (D) (Scale bar: 20 μm).

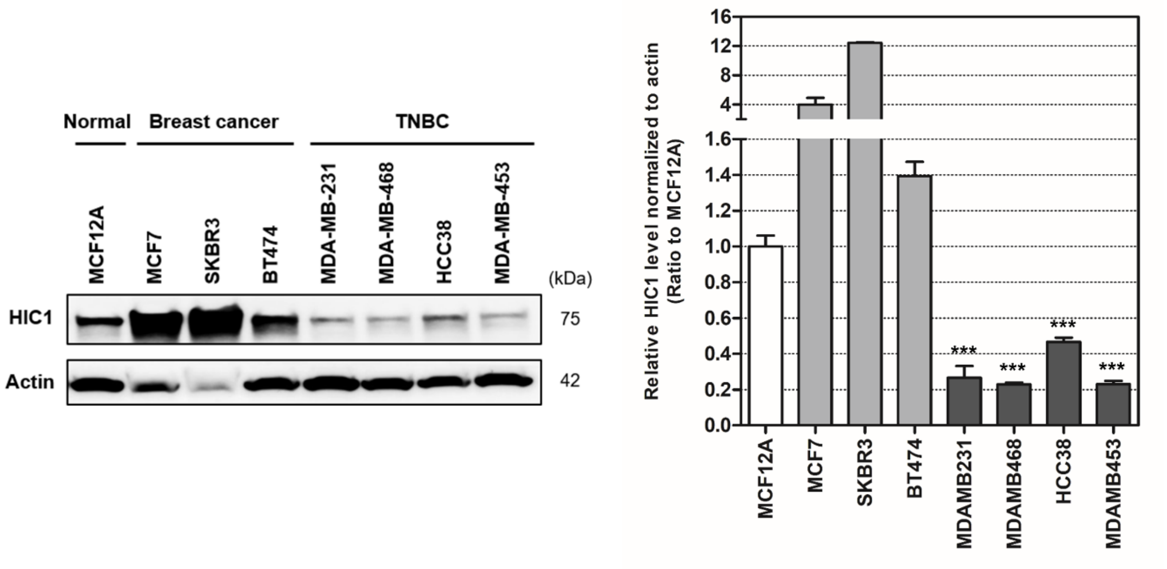

Figure S6. HIC1 protein expression levels of various breast cell lines. Western blot analysis of HIC1 protein expression levels in breast cancer cell lines (MCF7, SKBR3, BT474, MDA-MB-231, MDA-MB-468, HCC38, and MDA-MB-453) compared with a human normal breast cell (MCF12A). Mean ± SEM, $n = 3$. $^{***}p < 0.001$ *vs.* HIC1 expression in MCF12A.

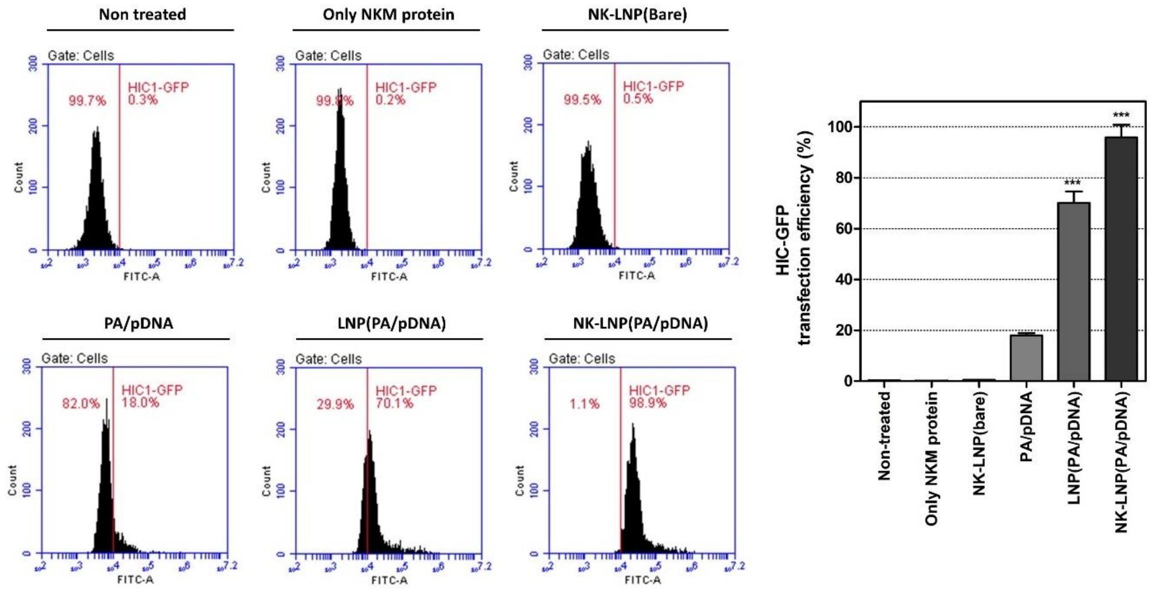

Quantitative analysis of transfected HIC1 protein. The presented results are the FACS data obtained under the same conditions as the WB results in Figure 3A. The medium was replaced 6 hours after treatment for each sample, and FACS analysis was performed 72 hours later. Here, we wanted to distinguish it from the slightly expressed HIC1 protein in TNBC cells. Therefore, we used a gene with GFP attached to the C-terminal of HIC1

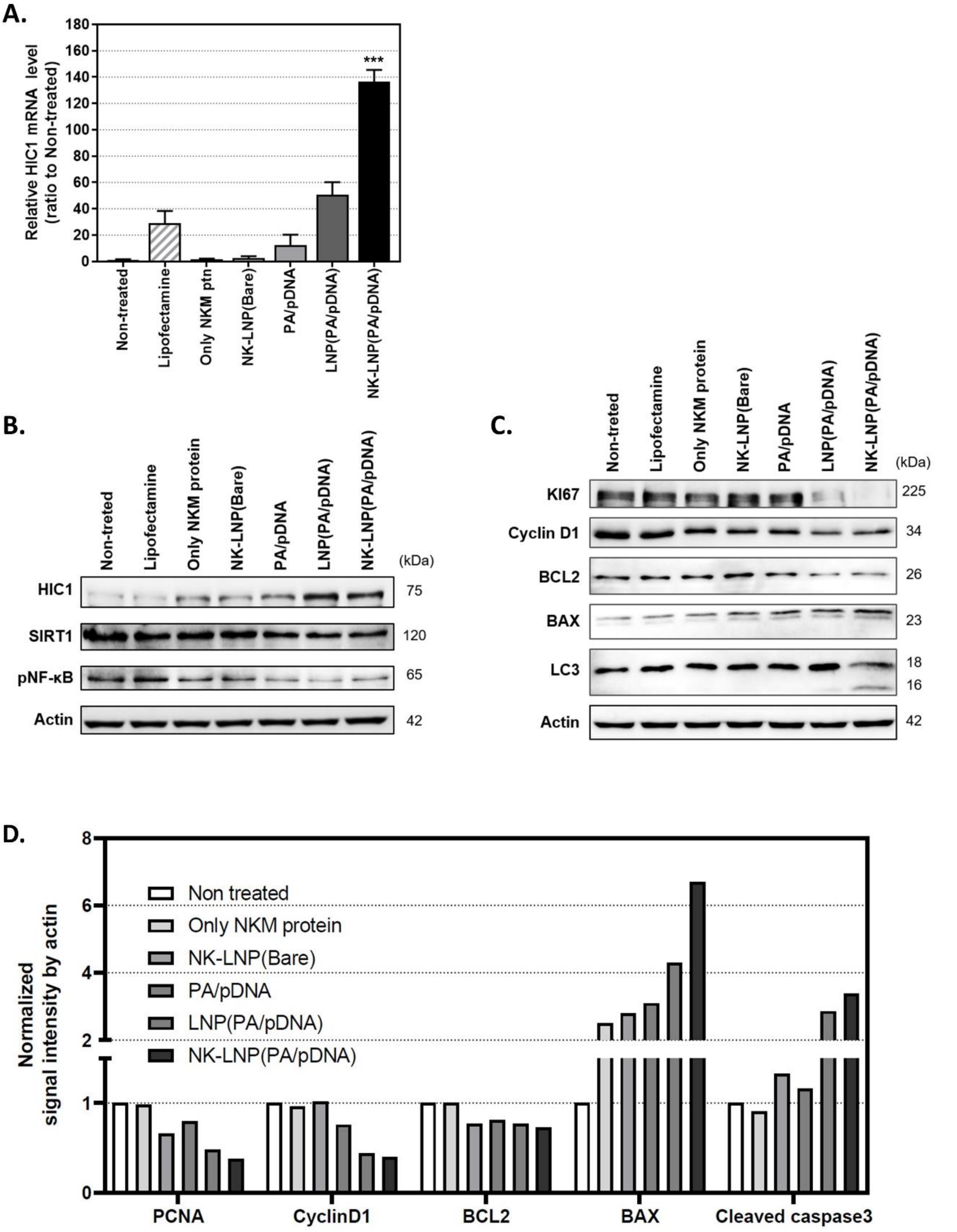

Figure S7. Western blot analysis after treatment of various materials in MDA-MB-468 cells. (A) Relative *HIC1* mRNA level after treatment in each group by qRT-PCR. Mean ± SEM, $n = 3$. $^{***}p < 0.001$ *vs.* non-treated. (B) Western blot analysis of restoring *HIC1* downstream genes (*HIC1*, *SIRT1*, and *phospho-NFκB*). (C) Western blot analysis of cell proliferation (Ki67), cell cycle (Cyclin D1), apoptosis (BCL2, BAX), and autophagy (LC3) genes. (D) quantification data of Figure 3E.

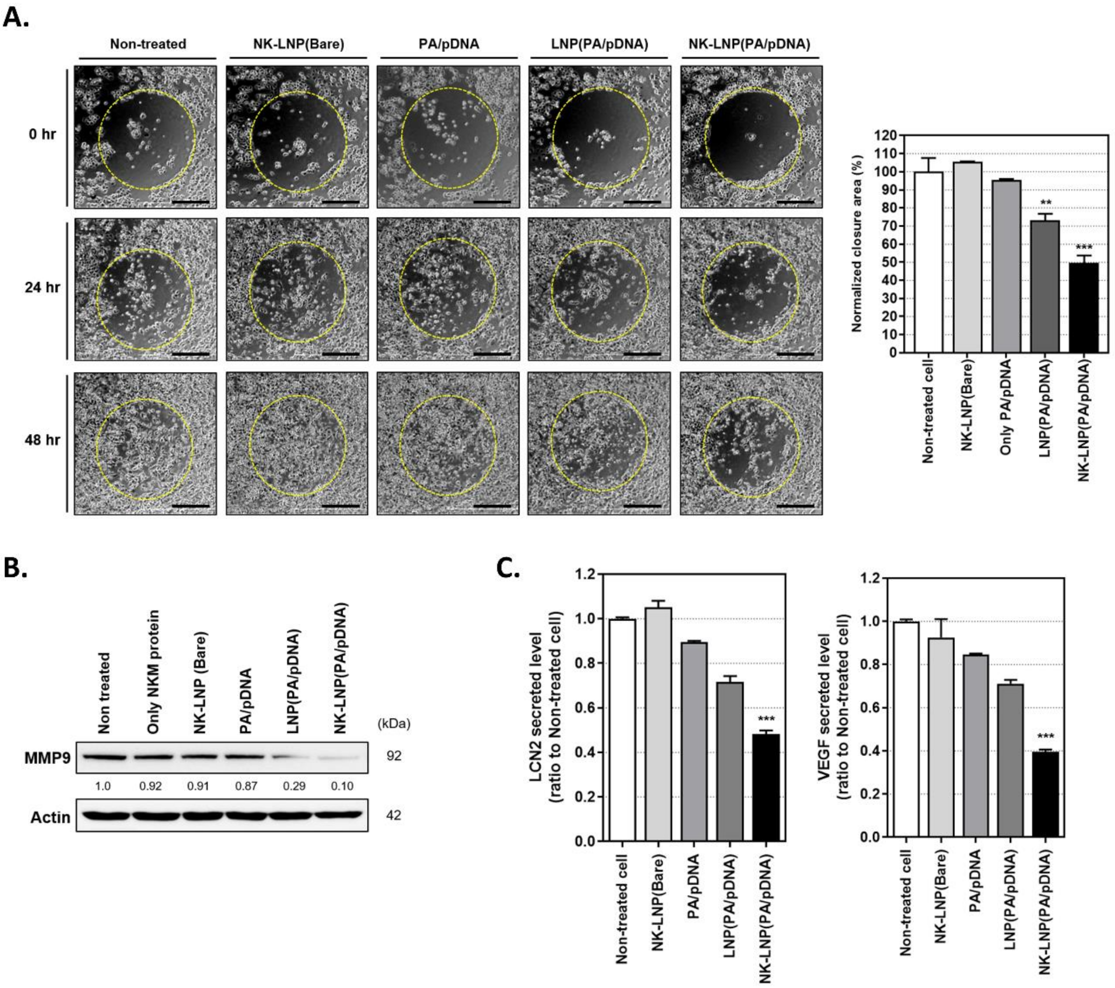

Figure S8. Metastasis inhibition assay of NK-LNP(PA/pDNA) without C18-ceramide in MDA-MB-468 cells. (A) Cell migration from each group by restoring HIC1 expression of NK-LNP(PA/pDNA). Mean ± SEM, n = 3. $^{**}p$ <0.01 and $^{***}p$ < 0.001 *vs.* non-treated cell (Scale bar: 20 μm). (B) Western blot of metastasis (MMP9)-related protein after various treatments. (C) Relative quantification of LCN2 and angiogenesis (VEGF) secreted level compared with non-treated cells by ELISA analysis. Mean ± SEM, n = 3. $^{***}p$ < 0.001 *vs.* non-treated cell.

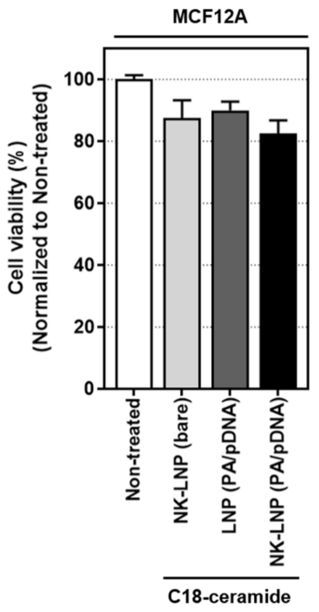

Figure S9. Cell viability assessment of NK-LNP(Bare), LNP(PA/pDNA), and NK-LNP(PA/pDNA) with C18-ceramide in MCF12A.

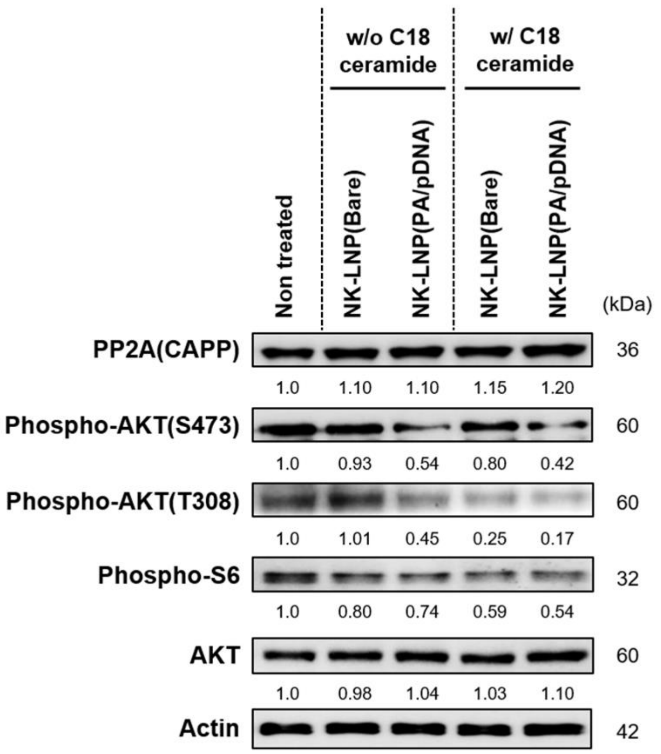

Figure S10. AKT pathway inhibition of MDA-MB-468 cells after NK-LNP(bare) and NK-LNP(PA/pDNA) with C18-ceramide treatments.

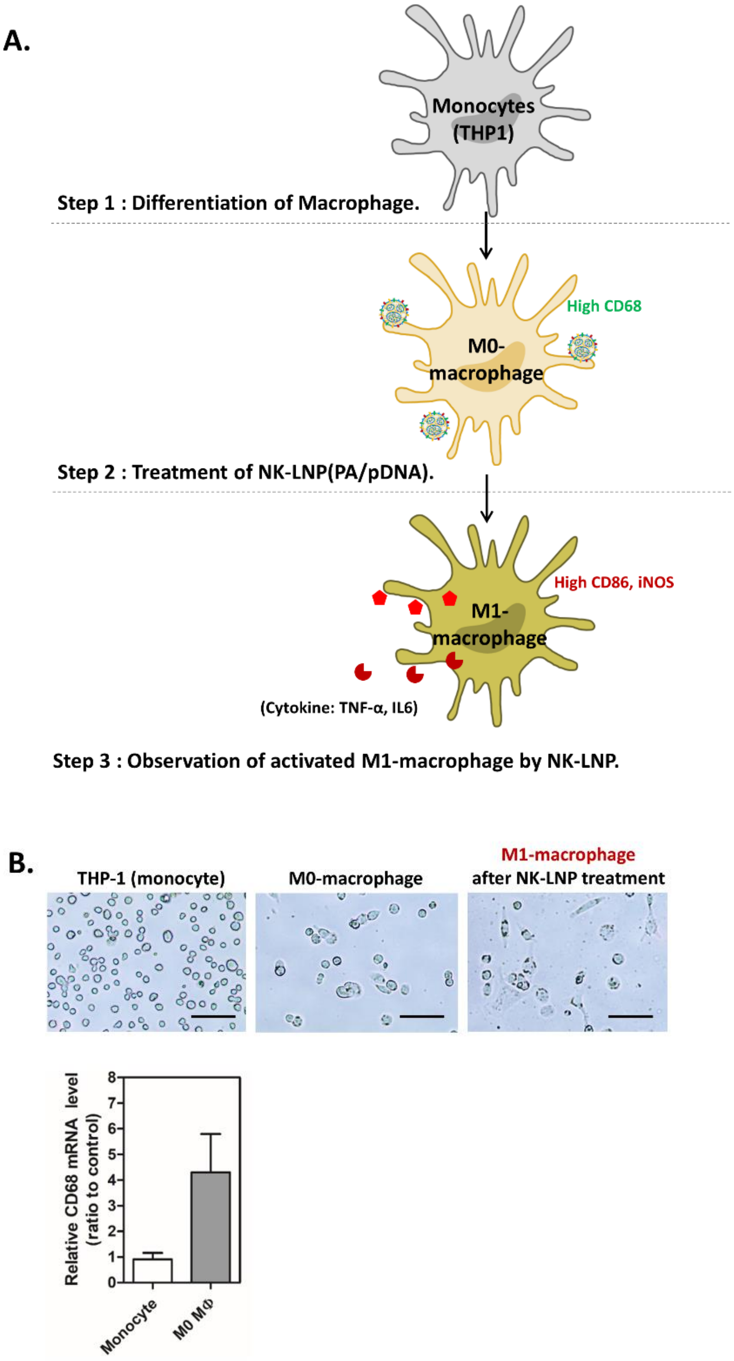

Figure S11. ***In vitro*** **M1-macrophage polarization after NK-LNP(PA/pDNA) treatment to THP-1 cells.** (A) Schematic illustration of polarized M1-macrophage by NK-LNP(PA/pDNA). (B) Cell morphology of THP-1 (monocytes), PMA treated-M0 macrophage, and M1 macrophage cells by light microscopy (Scale bar: 20 μm). Polarized macrophage confirmation with and without PMA treatment (M0-macrophage) by assessing relative CD68 mRNA levels, respectively.

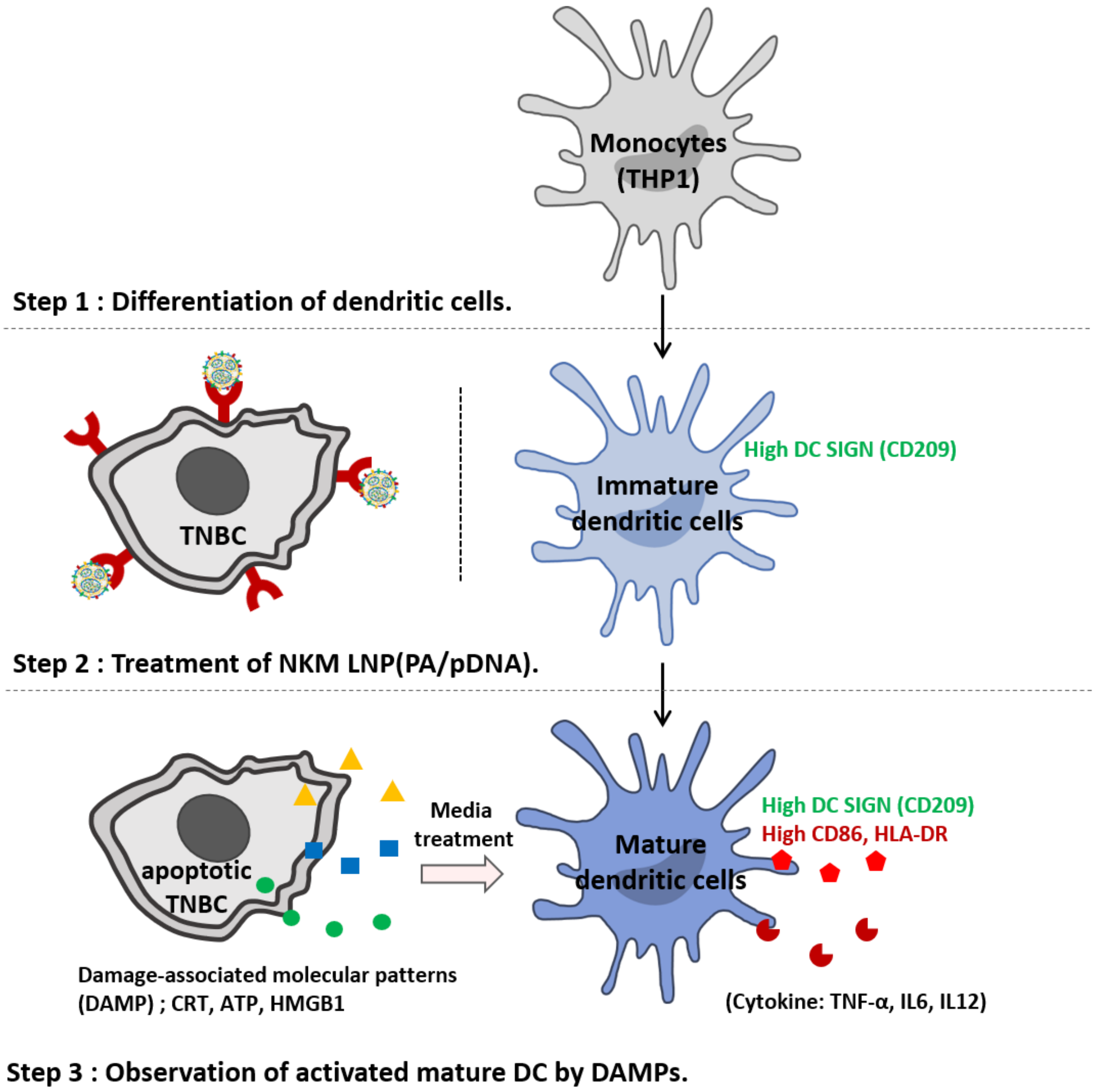

Figure S12. Schematic illustration of *in vitro* dendritic cell differentiation by NK-LNP(PA/pDNA) in THP-1 cells. THP-1 cells (monocytes) were differentiated into immature dendritic cells by human recombinant IL4 and GM-CSF protein for 5 days before TNBC treatment with NK-LNP(PA/pDNA). After that, immature dendritic cells were co-cultured with NK-LNP(PA/pDNA) treated TNBC. Eventually, TNBC cells showed apoptosis induced by NK-LNP(PA/pDNA) and secreted DAMP (damage-associated molecular pattern). These DAMPs were consequently differentiated mature dendritic cells (mDC). These mDCs then secreted inflammatory cytokines (TNFα, IL6, and IL12) to affect anti-tumor immunity.

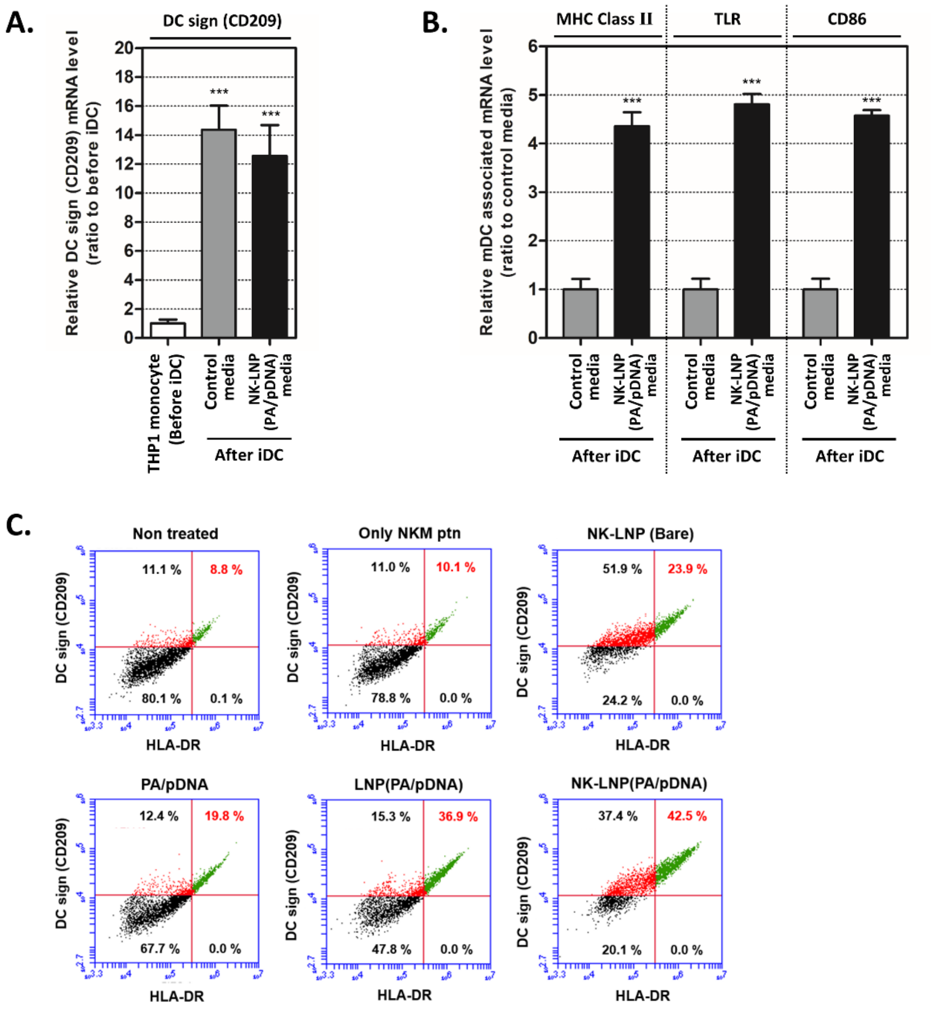

Figure S13. ***In vitro*** **dendritic cell differentiation by NK-LNP(PA/pDNA) in THP-1 cells.** (A) Confirmation of differentiated immature dendritic cells (iDC) by assessment of relative DC sign (CD209) mRNA level in THP1 cells. Gene expression by qRT-PCR (B) and FACS analysis (C) of differentiated mature dendritic cells (mDC) by DAMP. DAMP was secreted by apoptotic TNBC by NK-LNP(PA/pDNA). Mean ± SEM, $n = 3$. $^{***}p < 0.001$ *vs.* control media

CD68	(F) 5'-CGAGCATCATTCTTTCACCAGCT-3',<br>(R) 5'-ATGAGAGGCAGCAAGATGGACC-3' (origene, #HP226449)
iNOS	(F) 5'-GCTCTACACCTCCAATGTGACC-3',<br>(R) 5'-CTGCCGAGATTTGAGCCTCATG-3' (origene, #HP200591)
CD206	(F) 5'-AGCCAACACCAGCTCCTCAAGA-3',<br>(R) 5'-CAAAACGCTCGCGCATTGTCCA-3' (origene, #HP206121)
CD163	(F) 5'-CCAGAAGGAACTTGTAGCCACAG-3',<br>(R) 5'-CAGGCACCAAGCGTTTTGAGCT-3' (origene, #HP207589)
DC sign (CD209)	(F) 5'-GCAGTCTTCCAGAAGTAACCGC-3',<br>(R) 5'-GCTCTCCTCTGTTCCAATACTGC-3' (origene, #HP214086)
HLA-DR	(F) 5'-GAGCAAGATGCTGAGTGGAGTC-3',<br>(R) 5'-CTGTTGGCTGAAGTCCAGAGTG-3' (origene, #HP214086)
TLR	(F) 5'-CCCTGAGGCATTTAGGCAGCTA-3',<br>(R) 5'-AGGTAGAGAGGTGGCTTAGGCT-3' (origene, #HP226301)
CD86	(F) 5'-CCATCAGCTTGTCTGTTTCATTCC-3',<br>(R) 5'-GCTGTAATCCAAGGAATGTGGTC-3' (origene, #HP233662)
β-Actin	(F) 5'-ACGTGGACATCCGCAAAGA-3',<br>(R) 5'-CTCAGGAGGAGCAATGATC-3'

Table S1. List of primers used for real-time PCR.

**Recombinant human protamine amino acid sequence**
HHHHHHKETAAAKFERQHMDSARYRCCRSQSRSRYYRQRQRSRRRRRRSCQTRRRAMRCCRPRYRPRCRRHHHHHHH
**Recombinant human protamine nucleic acid sequence**
atggccaggtacagatgctgtcgcagccagagccggagcagatattaccgccagagacaaagaagtcgcagacgaaggaggcggagctgccagacacggaggagagccatgaggtgctgccgccccaggtacagaccgagatgtagaagacac
**Protamine sulfate (from salmon) amino acid sequence**
PRRRRSSSRPVRRRRRPRVSRRRRRRGGRRRR

Table S2. Amino acid and DNA sequence of recombinant human protamine (PA) and protamine sulfate (PS) in this study. (His-tag sequence, blue; S-tag sequence, green; and human protamine sequence, red).
